# Population structure, seasonal genotypic differentiation, and clonal diversity of weedy dandelions in three Boston area populations (*Taraxacum* sp.)

**DOI:** 10.1002/ece3.7870

**Published:** 2021-07-28

**Authors:** Lisa Mazumder, Rick Kesseli

**Affiliations:** ^1^ Department of Biology University of Massachusetts Boston Boston MA USA

**Keywords:** apomixis, clonal diversity, cpDNA, dandelion, linkage disequilibrium, microsatellites

## Abstract

Weedy dandelions have a worldwide distribution and thrive in urban environments despite a lack of sexual reproduction throughout most of its range. North American dandelions, introduced from Eurasia, are believed to be primarily, if not exclusively, apomictic triploids. In some European populations, apomicts co‐occur with diploid sexual individuals and hybridizations can create genetically unique apomicts, which may subsequently disperse and establish new populations globally. Using six nuclear microsatellite markers and a cpDNA intergenic spacer, we investigate the impact of this unusual natural history on population structure and diversity in three urban Boston area dandelion populations. Our results show high levels of genetic diversity within populations, spatial population structure, and seasonal genotypic differentiation in flowering times. We find evidence that sexual reproduction and recombination, presumably in Europe, and extensive gene flow drive these patterns of diversity and create the appearance of panmixia despite the lack of evidence for local sexual reproduction.

## Graphical abstract

This study investigates population genetics, clonal diversity, and genotypic differences in flowering time of weedy dandelions in an urban area. We find population structure among sites and that specific genotypes are flowering during certain seasons. Populations exhibit high genetic diversity, are asexual, and mimic panmixia as a result of mass dispersal from European source populations.

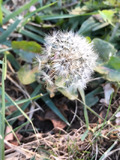

## 1 INTRODUCTION

Weedy dandelion, *Taraxacum officinale* Weber ex. Wiggers, is a highly successful, cosmopolitan, short‐lived perennial species that thrives in disturbed environments. Dandelion likely originated 30 million years ago in the western Himalayas, and its recent history and center of diversity is in western Asia and Europe (Solbrig, 1971). The now notorious weed, once revered for its nutritional and medicinal value, was carried both deliberately and passively to new regions by European settlers (Richards, 1973; Solbrig, 1971). More recently, air travel and agricultural transfer have likely increased dispersal frequency and extent for many plant propagules (Hodkinson & Thompson, 1997; Mack & Lonsdale, 2001). In addition to this human‐aided dispersal, the success of the dandelion has likely been facilitated by recently discovered novel airflow vortex behaviors associated with the pappus, resulting in dispersal distances reported to be as high as 150 km or more (Cummins et al., 2018 and citations there in).

Dandelion also has an unusual apomictic reproductive system, resulting in hundreds of genetically identical clonal seeds being produced from single triploid parents (Asker & Jerling, 1992). Though apomixis confers a highly efficient mode of reproduction, purely apomictic populations should suffer due to low genetic diversity and an accumulation of deleterious mutations (Felsenstein, 1974; Müller, 1964). However, diploid sexual and triploid apomictic dandelions can hybridize in the native European range where they coexist, thereby creating new, genetically unique apomictic lineages. These new apomictic lines then presumably disperse to establish new populations (Menken et al., 1995; Richards, 1973; Van Der Hulst et al., 2003). This mixed reproductive strategy allows the dandelion to generate new genetic diversity via sexual reproduction while taking advantage of the efficiency of apomictic reproduction. Though purely sexual populations are rare even in Europe (Menken et al., 1995; Van Der Hulst et al., 2003), cryptic sexual reproduction is certainly possible outside of Europe among pollen‐producing apomicts or with hidden sexual diploids and this would provide an evolutionary advantage, especially in the face of unexpected environmental stochasticity (Williams, 1975).

In the introduced range of North America, where only triploid apomictic dandelions have been reported, prior studies have uncovered higher than expected genetic diversity for an asexual species (King, 1993; Lyman & Ellstrand, 1984; Rogstad et al., 2001), with multiple clonal genotypes existing in the same area. Prior studies assessing genetic diversity, as well as a more recent study assessing genomic size variation in dandelions, have not identified any sexual dandelions in North America (Iaffaldano et al., 2017; King, 1993; Lyman & Ellstrand, 1984). The studies of Lyman and Ellstrand (1984) and King (1993) sampled a wide geographic range of North America with relatively low sample sizes per population and concluded that the observed genetic diversity was arising primarily via sexual recombination in European mixed apomictic–sexual populations and new apomictic lineages were dispersing over to North America. Rogstad et al. (2001) studied a more localized region with deeper sampling of three populations in Kentucky and Ohio using variable number tandem repeat (VNTR) loci. While this study demonstrated a strong role of sexual recombination in the observed genetic diversity, the researchers also suggested that somatic mutation may be playing a greater role in diversification in clonal lineages than previously thought, leading to slightly differentiated “clonal families.” The higher level of diversity in Rogstad et al. (2001) study probably reflects the nature of the VNTR molecular markers, which are highly polymorphic and have high mutation rates.

The occurrence of genetically distinct clones in populations begs the question about if and how these clonal lineages evolve and adapt to local conditions. This question is particularly intriguing in the face of urbanization and global climate change, as urban populations must have wide environmental tolerances and be able to survive extreme abiotic stresses (Johnson et al., 2015). Life history and flowering time differences among clonal genotypes may allow genetically diverse dandelions to coexist in the same locations. Seasonal variability across different geographic regions of North America affects the exact months during which dandelions experience flowering (Collier & Rogstad, 2004; Vellend et al., 2009). In a controlled greenhouse experiment, a significant difference in flowering time among clones was uncovered (Collier & Rogstad, 2004). Common garden studies also showed ecological differences in genotypes, including flowering time (Vellend et al., 2009). Shifting flowering times in the assessment of seasonality, particularly in urban environments where true seasonality is affected by urban structures, has recently become an important and new area of study. Studies show that the flowering seasons of many plant species have been shifting and extending, with both earlier and longer durations than ever before (Primack et al., 2004).

In this study, we investigate the local population genetics of Boston‐area, urban dandelions. Our research objectives are as follows: (1) to characterize clonal diversity in three populations using six polymorphic microsatellite markers and the trnH‐psbA intergenic spacer region of chloroplast DNA, (2) to assess spatial structure and flowering time patterns as an indicator of life history diversity, and (3) to test for linkage disequilibrium, the signature of clonal reproduction, and panmixia to reveal the relative importance of sexual reproduction, dispersal and somatic mutation in the establishment of local population structure.

## 2 MATERIALS AND METHODS

### 2.1 Sample collection

Sampling for this study was performed over two years on a monthly basis April through November in 2017 and 2018; plants flowering well beyond this period are found but flowering is sporadic and their numbers are low. We collected 568 dandelion samples from three stable, undisturbed (other than occasional mowing) sites covering approximately a 20 km range from north to the south of Boston, MA: Prospect Hill Monument, Somerville, MA; Cedar Grove Cemetery, Boston, MA; and 12 Moon Farm in Milton, MA, respectively. Leaf tissue was collected only from individuals in flower, separated at least four meters apart, and placed in separate Ziploc bags. Samples were kept fresh or stored in −20°C until the time of extraction. Leaf tissue was typically not stored in the freezer for longer than one month before DNA extraction.

### 2.2 DNA extraction, PCR, sequencing and fragment analysis

Approximately 100 mg of leaf tissue was used for DNA extraction using the DNeasy Plant Mini Kit (Qiagen, Valencia, CA) following manufacturer's protocol with slight modifications. Frozen leaf tissue was measured out and manually crushed using sterilized ceramic mortar and pestles. Lysation buffer (200 µl) was added directly onto the leaf tissue to aid in manual crushing. The elution buffer was heated at 65°C for 10 min prior to the final DNA elution step to increase DNA yield. Whole genomic DNA was quantified and checked for purity on a NanoDrop 2000C. DNA dilutions on samples were performed to a level of 0.5–0.7 ng. PCR was performed using the diluted DNA as the template. PCR for the trnH‐psbA intergenic spacer region was performed according to primers and PCR specifications from Hamilton (1999). PCRs for the cpDNA were performed in a total volume of 25 µl per reaction with 8.8 µl autoclaved MilliQ water, 5 µl of 5X PCR green flexi buffer, 0.5 µl of 10 mM dNTPs, 2.5 µl of 25 mM MgCl2, 1.5 µl of 10 µM of forward and reverse primers, 0.2 µl of GoTaq DNA polymerase (Promega), and 5 µl of diluted DNA. The thermal cycler conditions for the trnH‐psbA region were as follows: 95°C for 2 min; 30 cycles of 95°C for 1 min, 52°C for 1 min, 72°C for 1 min; ending with 72°C for 5 min, and a final 4°C hold. Amplicons for the trnH‐psbA region were confirmed on 1% ethidium bromide agarose gels, and positive PCR products were prepared and sent for PCR cleanup and Sanger sequencing in the forward direction to Eton Biosciences, Inc. Multiplex PCR was performed for six microsatellites developed specifically for dandelions (Falque et al., 1998; Vasut et al., 2004) in two multiplexed sets of three loci each using the fluorescent dyes 6‐FAM, VIC, AND NED (Thermo Fisher Scientific, Inc.). The fluorescent tag was directly applied to the 5′ end of the forward primer, while the reverse primer was kept untagged. The loci (6‐FAM)‐MSTA31, (VIC)‐MSTA67, and (NED)‐MSTA78 were multiplexed together; while the loci (6‐FAM)‐MSTA101, (VIC)‐MSTA102, and (NED)‐MSTA143 were multiplexed together. PCRs for the microsatellite markers were performed in a total volume of 25 µl per reaction with 9.8 µl autoclaved MilliQ water, 5 µl of 5X PCR green flexi buffer, 0.5 µl of 10 mM dNTPs, 2.5 µl of 25 mM MgCl2, 0.33 µl of 10 µM of forward and reverse primers (a total of 3 multiplexed pairs), 0.2 µl of GoTaq DNA polymerase (Promega), and 5 µl of diluted DNA. The thermal cycler conditions for touchdown PCR as described in Zavada et al. (2017) were followed for the amplification of the microsatellite markers. Microsatellite amplicons were confirmed with 2% ethidium bromide agarose gels. Multiplexed PCR products of the microsatellite markers were sent for fragment analysis to the Roy J. Carver Biotechnology center at the University of Illinois, Urbana‐Champaign where samples were run on an Applied Biosystems 3730xl DNA Analyzer.

### 2.3 Data processing and analysis

For the cpDNA, a randomized subset of 96 individuals spanning the three sites and all collection time intervals was sequenced in the forward direction for the trnH‐psbA marker; 93 high‐quality sequences were obtained from the sequencing effort. Sanger sequences of the cpDNA were edited for artifacts, and multiple alignment of sequences was constructed using the CLUSTALW algorithm performed using Geneious version 10.0.5 (Kearse et al., 2012). A BLAST was performed against the NCBI GenBank® database to confirm that sequences were from dandelion DNA. A FASTA file of the alignment was exported for use in downstream haplotype analysis in R version 3.4.4 (R Core Team). Haplotype networks were constructed using the R package *pegas* version 0.11 (Paradis, 2010).

For the microsatellite markers, 568 samples were assayed for fragment sizes. Peak profiles were visualized and manually scored using the microsatellite plug‐in in Geneious version 10.0.5 and stored in a csv file format. Allele bins were determined by observing common peak patterns and seasonal categories of spring (April–June), summer (July–August), and fall (September–November) were assigned based on the month of collection. The R package *poppr* version 2.8.3 (Kamvar et al., 2014, 2015) was used for calculation of population genetics statistics, linkage disequilibrium (LD), and gene flow analysis with k‐means hierarchical clustering. For LD, the index of association, $\bar{r}$_d_, and *p*‐value were calculated for each of the three populations. Calculations were performed for both the entire dataset as well as for clone‐corrected data in which duplicate individuals with identical alleles for all loci were removed. The null hypothesis for each test was that there would be no LD between markers. The R package *adegenet* version 2.1.1 (Jombart, 2008; Jombart & Ahmed, 2011) was used to perform Discriminant Analysis of Principal Components (DAPC) to infer population structure; by determining genetic clusters spatially by site, and temporally by season. The R package *ape* version 5.1 (Paradis & Schliep, 2019) was used to create and visualize the dendrograms in the gene flow analysis; genotypes with missing data were removed before generating the dendrograms or k‐means clusters.

An analysis of molecular variance (AMOVA) was performed in the R package *poppr* version 2.8.0 (Kamvar et al., 2014, 2015) with significance testing of the variance matrices via random permutations (Excoffier et al., 1992) performed with R package *ade4* version 1.7.11 (Bougeard & Dray, 2018; Chessel et al., 2004; Dray & Dufour, 2007; Dray et al., 2007; Thioulouse et al., 2018). AMOVA was performed for both non‐clone‐corrected and clone‐corrected data. Pairwise G_st_ values (Hedrick, 2005) were calculated for the three populations and the three seasons; as well as separate populations and seasons using the R package *poppr* version 2.8.0 (Kamvar et al., 2014, 2015). For the assessment of clonal diversity in the populations, a customized R script was used to bin alleles when the number of alleles was identical for each of the respective loci but differed in length by ±4 base pairs. Based on allele pattern recognition, it was assumed that those differing in length by a few base pairs but containing exactly the same number of alleles at all the positions for each of the six loci are likely the same clone and the slight base pair difference can be attributed to scoring bias or software error.

A Bayesian clustering approach, STRUCTURE version 2.3.4 (Pritchard et al., 2000; Falush et al., 2003, Falush et al., 2007; and Hubisz et al., 2009), was applied to the microsatellite data. Analyses were run for the total dataset of *n* = 551 for the three sites set as “populations” as well as the three seasons set as the “populations.” Two additional analyses were performed: (1) with the cpDNA haplotype groupings set as “populations” for *n* = 91 samples in order to visualize nuclear–cytoplasmic recombinant groupings established at each population site and (2) with the clone‐corrected dataset where all multilocus genotypes were represented once and any samples with missing data removed for *n* = 270. For all STRUCTURE analyses, admixture model and correlation between alleles were assumed. For the total dataset of *n* = 551, an initial simulation was performed for K = 2 through K = 20 for burn‐in period of 200,000 with an additional 200,000 Markov Chain Monte Carlo (MCMC) replicates over five iterations in order to implement post hoc tests of optimal K clusters. For both the haplotype subset of *n* = 91 and clone‐corrected subset of *n* = 270, an initial simulation of K = 2 through K = 15 for burn‐in period of 200,000 with an additional 200,000 MCMC replicates over five iterations was performed. These initial simulations were run through Structure Harvester (http://taylor0.biology.ucla.edu/structureHarvester/; Earl & vonHoldt, 2012) where optimal number of clusters, K, were determined from “Delta K’’ computed based on rate of change of the mean of the standardized log‐likelihood (Evanno et al., 2005). For the total dataset of *n* = 551, the optimal was K = 11; in the haplotype subset of *n* = 91, the optimal was K = 3; and in the clone‐corrected subset, the optimal was K = 12. Using these optimal K values, a subsequent simulation was performed for each dataset for burn‐in period 200,000 and 1,000,000 MCMC replicates.

## 3 RESULTS

### 3.1 cpDNA analysis and haplotype networks

**FIGURE 1:**
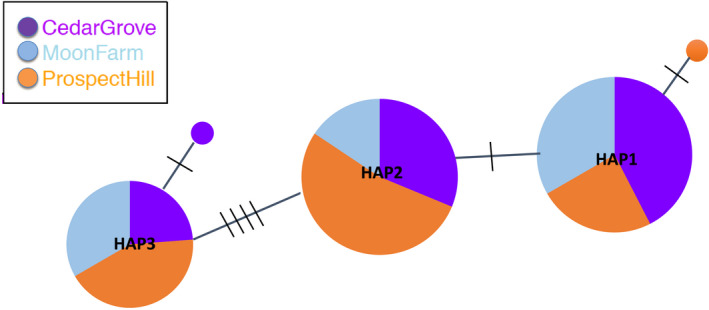
Haplotype network of the trnH‐psbA cpDNA marker of 93 samples shows three major haplotypes with 21–33 individuals; and two uncommon haplotypes with one individual each. Hatch marks indicate number of SNPs or indels between haplotypes

We obtained 93 high‐quality sequences from the sequencing effort. Sequence length of the multiple alignment is 410 bp. Haplotype network analysis in the R package *pegas* (Paradis, 2010) resulted in five total haplotypes, with 92.5% of samples falling into three main haplotypes. HAP1 and HAP2 each have 33 and 32 individuals respectively, while HAP3 has 21 individuals. Two uncommon haplotypes had one sample each. The three common haplotypes were detected in all sites, while one uncommon haplotype was from Cedar Grove Cemetery and one from Prospect Hill Monument (Figure 1).

### 3.2 Allelic diversity of nuclear markers, population and seasonal structure

The six microsatellite markers showed substantial variability with 14–39 alleles, high levels of genetic diversity (Hexp = 0.77–0.94), and evenness (E_5_ = 0.59–0.73) making these markers very useful for our study (Table 1). Both Nei's genetic diversity (Hexp) and Simpson index, 1949 (1‐D), were calculated and both produced equal results and we are only reporting Hexp herein. Furthermore, there are an enormous number of different multilocus genotypes detected. Of the 551 individuals genotyped, 330 were different and between 95 and 124 genotypes were identified in each site (Table 2). A calculation of pairwise G_st_ between the three populations showed the least differentiation between PH and CG (G_st_ = 0.018) and more differentiation between MF and PH (G_st_ = 0.049) and MF and CG (G_st_ = 0.049). Pairwise G_st_ calculated between the seasons showed the least differentiation between spring and summer genotypes (G_st_ = 0.004); followed by Summer and Fall (G_st_ = 0.010); the most differentiation between spring and fall genotypes (G_st_ = 0.046).

**TABLE 1:** Statistics for each of the six microsatellite loci utilized in this study

Locus	# observed alleles	Nei’s, 1978 gene diversity (Hexp)	Evenness (E_5_)
MSTA31	29.00	0.89	0.71
MSTA67	39.00	0.94	0.73
MSTA78	26.00	0.81	0.62
MSTA101	22.00	0.82	0.61
MSTA102	23.00	0.78	0.59
MSTA143	14.00	0.77	0.69
Mean	25.50	0.83	0.66

**TABLE 2:** Population statistics

Pop	*N*	MLG	N_c_	MLG_c_	eMLG	SE	H	G	lambda	E.5	Hexp
PH	192	124	156	90	96.9	2.92	4.44	46.4	0.978	0.541	0.829
CG	216	148	184	116	104.1	3.28	4.71	72.4	0.986	0.649	0.834
MF	143	95	116	69	95.0	0.00	4.14	28.3	0.965	0.443	0.820
Total	551	330	456	256	105.8	4.59	5.28	79.2	0.987	0.401	0.834

Note

*N* is number of samples; MLG is number of observed multilocus genotypes of the total dataset of *n* = 551. N_c_ and MLG_c_ refer to the subset of samples with complete six‐locus genotypes and no missing data. All subsequent statistics in the table are calculated only for *n* = 551, where eMLG is expected number of multilocus genotypes at the smallest sample size ≥10 based on rarefraction; SE is standard error based on eMLG; H is Shannon–Wiener (1948) on MLG diversity; G is Stoddart & Taylor’s, 1988 index of MLG diversity; lambda is Simpson’s, 1949 index; E.5 is evenness; Hexp is Nei’s, 1978 unbiased gene diversity.

**FIGURE 2:**
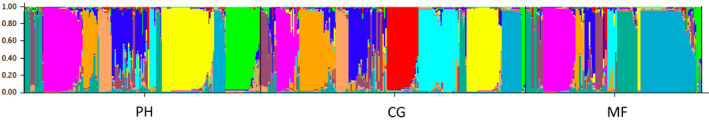
STRUCTURE assignment plot of the total dataset of *n* = 551 based on the six SSR markers with K = 11 showing some population‐level genotypic specificity in the three sites (PH, Prospect Hill; CG, Cedar Grove; MF, Moon Farm), but also evidence of admixture

**FIGURE 3:**
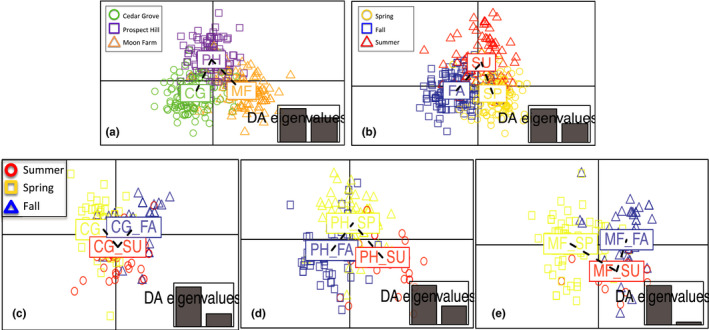
(a) Discriminant analysis of principal components (DAPC) of the six SSR markers by population site show population structure with some overlap between sites. (b) DAPC shown by season for all populations (FA, fall; SU, summer; SP, spring) shows strong separation between genotypes flowering in fall and spring with some separation, but more overlap of genotypes flowering in the summer. (c–e) DAPC by season for each population show a consistent separation of genotypes flowering in spring, summer, and fall

Bayesian STRUCTURE analyses and AMOVA all detected widespread diversity and population structure among the three sites. While STRUCTURE analyses may not be the most appropriate tool for clonal populations (Grünwald et al., 2017), it did clearly show that all populations had essentially pure genotypes from all K groups regardless of the K value (K = 3–12, with K = 11 being selected as the optimal value for the total dataset of *n* = 551) chosen, suggesting substantial seed flow of clones into sites. Some multilocus genotypes (clones) are found in all three populations but many are specific to populations, and the frequency of the genetic groups varied among sites (Figure 2). The DAPC analyses (Figure 3a) graphically shows this structure among sites. Seasonal clustering is also evident in the DAPC with genetically similar individuals flowering at similar times of year (Figure 3b). The seasonal analyses conducted for each of the three sites separately showed that true seasonal patterns were not confounded by location (Figure 3c–e). Finally, the AMOVA using clone‐corrected data confirmed many of these patterns, with observed Φ values falling outside the distributions expected from the permutations at all levels of analysis with substantial diversity partitioned among populations (*p* < .05) as well as among seasons within populations (*p* < .001).

**FIGURE 4:**
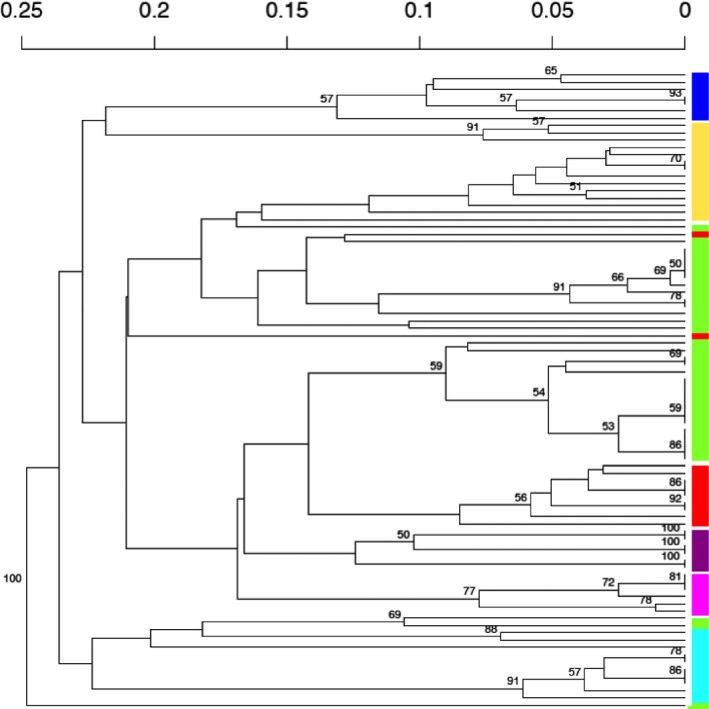
A randomized subset of 88 Cedar Grove samples shown in bootstrapped dendrogram using Bruvo's distances, a genetic distance metric for microsatellite markers. Colored leaves on the tree indicate eight k‐means clusters which do not correspond perfectly with the dendrogram clusters indicating panmixia and gene flow from other sources. The full datasets of Cedar Grove, as well as Prospect Hill and Moon Farm (not pictured), show similar results

We used a K‐means hierarchical clustering approach that has been used to investigate and differentiate the structure of clonal and random mating populations (https://grunwaldlab.github.io/Population_Genetics_in_R/Pop_Structure.html), to assess the fit of our dandelion data to these models (Figure 4). The bootstrapped dendrograms constructed from Bruvo's distances showed long branches generally indicative of more random mating populations. While we only show the dendrogram for a randomized subset of the Cedar Grove population for visualization purposes, this pattern of deep branching and mismatches is seen in the full datasets of each population as well. The assigned colored k‐means clusters (K = 8) were often misaligned with dendrogram branches in all analyses which is the expectation for more random mating and panmictic populations (Figure 4). Panmixia seems more pronounced in the Cedar Grove and Prospect Hill populations, and much less so in the Moon Farm population with most k‐means clusters grouping together with the bootstrapped dendrogram groupings.

### 3.3 Linkage disequilibrium

In the tests for each of the populations of the six nuclear microsatellite loci, for both the full and the clone‐corrected data, the null hypotheses of no LD were rejected (Table 3). The null hypotheses were also rejected for the combined analysis of all populations. As the null hypothesis of no linkage between is rejected for all three populations, it is suggestive of asexual reproduction as the prevalent mode of reproduction in these sites. Surprisingly however, the haplotypes identified from the chloroplast data are not associated with specific nuclear genomes as might be expected if asexual reproduction is the only mode of reproduction in these populations.

**TABLE 3:** Linkage disequilibrium (LD) summary for each of the populations for both non‐clone‐corrected (Non‐CC) and clone‐corrected (CC) data

Population	Clone correction	*N*	$\bar{r}$_d_	*p*‐value
CG	Non‐CC	216	0.152	.001
CG	CC	176	0.132	.002
MF	Non‐CC	143	0.258	.001
MF	CC	105	0.153	.001
PH	Non‐CC	192	0.205	.001
PH	CC	144	0.156	.001

### 3.4 Clonal diversity and differentiation

**FIGURE 5:**
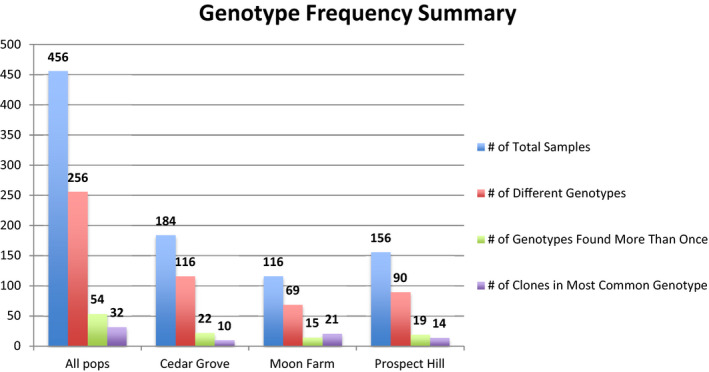
Histogram showing genotype frequencies summarizing number of total samples, number of different genotypes, number of genotypes found more than once, and number of clones present in the most common genotype found in all three populations (All pops) and for the three separate populations of Cedar Grove, Moon Farm, and Prospect Hill

Samples with missing data at one or more of the microsatellite loci were removed before clone counting, with 456 out of the 551 samples included in the final clonal diversity assessment. Clone counting was performed for the total dataset (All pops) as well as for each of the three populations separately. There were 256 different genotypes (56%) among the 456 samples, 202 being detected once only and 54 genotypes occurring more than once; the most frequent genotype was detected 32 times with 21 individuals in Moon Farm, 8 in Cedar Grove, and 3 in Prospect Hill. The proportion of different genotypes among the samples was similar for each site; 90 in 156 samples for Prospect Hill, 116 in 184 for Cedar Grove, and 69 in 116 for Moon Farm (Figure 5).

The four largest clones with 17–32 individuals sampled throughout the study were detected in all three populations. They each had distinct flowering time patterns, however. The largest clone flowered primarily in Fall of both years (66%), while the second and third largest clones with 28 and 26 samples, respectively, both flowered primarily in the spring of both years (86% and 69% respectively). The fourth largest clone with 17 individuals flowered about equally in fall and spring of 2017, but was not detected in any population in 2018.

Many of the 256 genotypes share common alleles in some loci but have different alleles at other loci, suggesting recombination as the source of genetic diversity. Indeed, in the entire dataset, there is only one pair of different clones that differs by a single allele at a single locus. All other clones differ from each other by multiple alleles at multiple loci, a pattern of variation not expected by local somatic mutation.

Further, a careful analysis of the STRUCTURE output showed that regardless of the assumed number of genetic groups, approximately 10% of the individuals in the entire population are the result of admixture (arbitrarily defined as individuals with less than 80% of their genome coming from one source). For the clone‐corrected analyses, 22%–32% of the multilocus genotypes are defined as having mixed ancestry. This reflects the role of sexual reproduction and recombination in the history of these individuals, though the timing, in Europe or in North America, of this is unknown. We addressed this timing issue, by examining the level of mixed ancestry as determined from the STRUCTURE analysis, with the prevalence of the various clones. From the clone‐corrected data in which each multilocus genotype is represented once in the analysis, none of the 15 largest clones represented by 5–32 individuals/clone in the full population have mixed ancestry at K = 12. These large clones are defined as >90% pure in the analysis. However, 32% of the genotypes are identified as having mixed ancestry (<80% of their genome coming from a single source) possibly suggesting that recombinants are more recent arrivals or evolved locally.

## 4 DISCUSSION

Apomixis is a rare and specialized form of asexual reproduction in plants that bypasses the need for fertilization and produces clonal seeds. Theoretically, genetic diversity in apomictic species, as with asexuality in general, should be very low and subject to the accumulation of deleterious mutations (Müller, 1964). This inability to generate new genetic diversity through sexual recombination may be unfavorable in stochastic and unstable environmental conditions (Williams, 1975). Remarkably, most apomictic plant species retain some mixture of sexual and asexual members, which can override the problem of low genetic diversity while also taking advantage of the efficient asexual reproductive strategy (Hojsgaard & Hörandl, 2015). As is the case in weedy *Taraxacum* species, closely related sexual members coexist in certain populations mainly in some areas of Europe and gene flow between apomictic and sexual types is possible, leading to newly formed and genetically unique apomicts (Hughes & Richards, 1988; Menken et al., 1995; Van Der Hulst et al., 2003). These genetically diverse apomictic dandelion lines then disperse and establish new populations elsewhere, such as throughout the introduced range of North America. In this interesting dichotomy, the rewards of both asexual and sexual reproduction are reaped: efficient proliferation and spread of genetic varieties as given by apomixis, and the continual introduction of new genetic varieties formed by sexual reproduction.

Apomictic species often have populations composed of many multilocus genotypes (Ellstrand & Roose, 1987), and *Taraxacum* follows this pattern as well. Remarkable, however, is that this truly cosmopolitan taxon seems to be sexual only in restricted regions of Europe. Prior studies encompassing the continent of North America, a region where only triploid apomicts are known to occur, Lyman and Ellstrand (1984) found between 1 and 13 different genotypes co‐occurring in the same field; King (1993) found 46% of her samples collected from across the continent were different genotypes using cpDNA and ribosomal DNA restriction enzyme analysis. Rogstad et al. (2001) conducted a study using highly polymorphic variable number tandem repeat (VNTR) markers on a more localized scale in three sites in Ohio and Kentucky and found a range of 19% clonal in the long transect to 34% clonal in the short transect, suggesting greater diversity than the studies of Lyman and Ellstrand (1984) and King (1993) even when conducted on a more localized scale. Our study contributes to this body of work by taking a deep sampling approach over an extended temporal scale in three local areas. Given the asexual breeding system of this species, previous studies probably assumed with good reason that dense local sampling might not reveal much additional variation. Our study did, however, identify very high levels of genetic diversity and also distinct population structure and genotypic changes associated with flowering time.

We detected three major cpDNA haplotype lineages, consistent with King (1993) in her North American study, but we also recorded high levels of nuclear diversity, identifying hundreds of unique genotypes and no cases of single genotypes for any sampling date at any site as reported in some studies. Several features of our study may have aided our efforts to reveal this extensive diversity, population structure, and life history (flowering time) differences. First, we chose “old,” well‐established sites that have not had major habitat disturbances in recent times (other than regular mowing). The populations of dandelions have probably been in these areas for decades if not longer and not recently established by a few founders. Second, our sampling took place over two years and across many months in each year. Third, our sites are within the greater metropolitan area surrounding Boston, which could enhance human‐aided dispersal. This experiment was not designed to look at different types of populations, but it is noteworthy that Moon Farm, the one private site and with infrequent mowing, was the most genetically distinct site (pairwise G_st_) and showed a less distinct signature of panmixia than the other two populations. These sampling strategies allowed us to assess the full array of genotypes in the area and the cumulative effects of population‐level processes.

While our study and others, together suggest that populations of *Taraxacum* in North America have high diversity and high numbers of different genotypes, the source of that variation is not as clear. Rogstad et al. (2001) did show a substantial number of unique clones within sites as our study did, but VNTR markers used in that study are more difficult to interpret. They generally concluded that much of the variation was probably caused by somatic mutations in clonal lineages. This is not true in our study. Our study using the SSRs suggests that the origin of the variation is recombination. Our loci are defined by multilocus and multiallelic banding patterns and clones possessed different combinations of these multiallelic loci, not single allele changes, a phenomenon also noted by Van Der Hulst et al., 2003 in their study of northern European apomictic populations. As noted previously, within the entire study that identified 256 different clones, only one pair of different clones differed by a single allele at a single locus. This one pair could conceivably be derived from a local somatic mutation (the pair of clones were both found in the Prospect Hill population). All other clones differed by multiple alleles at multiple loci. The pattern is much more akin to reticulate evolution resulting from hybridization (via sexual reproduction). King (1993) does note the lack of complete linkage disequilibrium between the nuclear and cpDNA genomes, and states that this must be from sex and hybridization, likely among lineages in Europe, which then disperse to North America. Our study shows this more clearly, with decoupled chloroplast and nuclear genomes as well as mismatches between the hierarchical genetic assignments and dendrogram relationships of genotypes giving populations a panmictic appearance (Figure 4). As expected however, LD is still substantial in all populations. Clearly, this high level of genetic diversity would require extraordinarily high seed dispersal if sexual reproduction is confined to Europe. Maybe not surprisingly, it appears that dandelions have just such an extraordinary flight mechanism as recently described by Cummins et al. (2018). Van Der Hulst et al. (2003) came to a similar conclusion that their northern European apomictic population was largely composed of genetic recombinants. However, it is likely that the source populations containing sexual members are much closer to their population than for our Boston area populations, again highlighting role of strong dispersal in mimicking the appearance of panmixia.

We also detected population structure and genotypic differences in flowering time within sites suggesting some local adaptation of clonal apomicts, though genetic drift despite the high dispersal may also contribute to the observed patterns. The spatial structure (3A) seemed surprising given the proximity of these sites, the prevalence of dandelions throughout the region, and the enormous level of gene flow via seed needed to generate the levels of diversity seen. The genetic structure in flowering times (Figure 3b) suggests that clones have diverse life history strategies and may be partitioning the available resources in sites.

While the prevailing view is that all genetic diversity is generated in Europe and dispersed to North American, we cannot discount the possibility of some cryptic local sexual recombination and its possible contribution to the genotypic diversity found in these populations. One interesting finding was that the 15 largest, most frequently occurring clones with 5–32 sampled individuals per genet, were never recombinant or of mixed ancestry based on STRUCTURE assignment analyses, suggesting that frequency may be correlated with time since establishment. This may indicate that recombinants are being generated in, and continuously dispersed from, Europe. Alternatively, this pattern might be achieved by dispersal and establishment of older clones from Europe in the past, followed by local cryptic recombination. Regardless, the propensity for dandelions to thrive in disturbed habitats created by humans makes this study system ripe for exploring urbanization, global climate change and local adaptation or selection of lineages. Further investigations of environmental conditions within each of the sites may reveal the factors that drive the establishment of population structure.

## CONFLICT OF INTERESTS

The authors report no competing interests.

## AUTHOR CONTRIBUTION

**Lisa Mazumder:** Conceptualization (equal); Data curation (lead); Formal analysis (lead); Investigation (equal); Methodology (equal); Validation (equal); Visualization (lead); Writing‐original draft (lead); Writing‐review & editing (equal). **Rick Kesseli:** Conceptualization (equal); Data curation (supporting); Formal analysis (supporting); Funding acquisition (lead); Investigation (equal); Methodology (equal); Project administration (lead); Resources (lead); Supervision (lead); Validation (equal); Visualization (supporting); Writing‐original draft (supporting); Writing‐review & editing (equal).

5

### DATA AVAILABILITY STATEMENT

Chloroplast DNA sequences of the trnH‐psbA intergenic spacer are available in GenBank^®^ (accessions MW451112‐MW451204). Microsatellite data as well as additional figures not pictured herein are available at 10.6084/m9.figshare.14544672.
